# Hypersensitivity reactions to contrast media: Part 2. Prevention of recurrent hypersensitivity reactions in adults. Updated guidelines by the ESUR Contrast Media Safety Committee

**DOI:** 10.1007/s00330-025-11676-0

**Published:** 2025-05-27

**Authors:** Aart J. van der Molen, Annick A. J. M. van de Ven, Francisco Vega, Ilona A. Dekkers, Remy W. F. Geenen, Marie-France Bellin, Michele Bertolotto, Torkel B. Brismar, Olivier Clément, Jean-Michel Correas, Katerina Deike, Gertraud Heinz, Andreas H. Mahnken, Carlo A. Mallio, Carlo C. Quattrocchi, Alexander Radbruch, Peter Reimer, Giles Roditi, Laura Romanini, Carmen Sebastià, Fulvio Stacul

**Affiliations:** 1 Department of Radiology, Leiden University Medical Center, Leiden, The Netherlands; 2 Department of Internal Medicine, Division of Allergology, University Medical Center Groningen, Groningen, The Netherlands; 3 Department of Allergy, Hospital Universitario de la Princesa, Madrid, Spain; 4 Department of Radiology, Amsterdam University Medical Center, Amsterdam, The Netherlands; 5 Department of Radiology, Northwest Clinics, Alkmaar, The Netherlands; 6 University Paris Saclay, AP-HP, University Hospital Bicêtre, Department of Radiology, BioMaps, Le Kremlin-Bicêtre, France; 7 Department of Radiology, Cattinara Hospital, University of Trieste, Trieste, Italy; 8 Department of Clinical Science, Intervention and Technology, Unit of Radiology, Karolinska Institute and Department of Radiology, Karolinska University Hospital in Huddinge, Stockholm, Sweden; 9 Université de Paris, AP-HP, Hôpital Européen Georges Pompidou, DMU Imagina, Service de Radiologie, Paris, France; 10 Université de Paris, AP-HP, Groupe Hospitalier Necker, DMU Imagina, Service de Radiologie, Paris, France; 11 Clinic for Diagnostic and Interventional Neuroradiology, University Clinic Bonn, and German Center for Neurodegenerative Diseases, DZNE, Bonn, Germany; 12 Athinoula A. Martinos Center for Biomedical Imaging, Massachusetts General Hospital, Charlestown, MA USA; 13 Department of Radiology, Landesklinikum St Pölten, St Pölten, Austria; 14 Department of Diagnostic and Interventional Radiology, Marburg University Hospital, Marburg, Germany; 15 Fondazione Policlinico Universitario Campus Bio-Medico, Roma, Italy; 16 Centre for Medical Sciences CISMed, University of Trento, Trento, Italy; 17 Department of Radiology, Institute for Diagnostic and Interventional Radiology, Klinikum Karlsruhe, Karlsruhe, Germany; 18 Department of Radiology, Glasgow Royal Infirmary, Glasgow, UK; 19 Department of Radiology, ASST Cremona, Cremona, Italy; 20 Department of Radiology, Hospital Clinic de Barcelona, Barcelona, Spain; 21 Department of Radiology, Ospedale Maggiore, Trieste, Italy

**Keywords:** Contrast media, Hypersensitivity, Recurrence, Prevention, Practice guideline

## Abstract

**Abstract:**

Hypersensitivity reactions to contrast media are infrequent and can occur either within the first 60 min following their intravascular administration (immediate reactions) or at a later time point (non-immediate reactions). Most hypersensitivity reactions are mild or moderate, while severe reactions are rare (less than 1 in every 10,000 administrations). After any moderate or severe immediate adverse reaction, serum tryptase must be measured within 1–4 h from the onset of symptoms and at least 24 h after symptoms have disappeared to confirm a hypersensitivity reaction. At least for all moderate-to-severe hypersensitivity reactions, the patient should be referred to a drug allergy specialist for an allergy evaluation with a panel of contrast media, and optionally, all hypersensitivity reactions when local drug allergy specialist capacity allows. Selecting an alternative contrast medium based on practical experience is challenging due to its high and variable cross-reactivity; therefore, the best option is to choose an alternative based on the results of an allergy evaluation. This approach is safer and more effective than premedication for preventing recurrent hypersensitivity reactions.

**Key Points:**

***Question*** *What is the optimal strategy in clinical practice to prevent recurrent hypersensitivity reactions in adults who previously experienced a hypersensitivity reaction to contrast media?*

***Findings*** *Serum tryptase should be measured within 1–4 h after a moderate or severe reaction, and at least all moderate or severe hypersensitivity reactions should be referred to an allergologist.*

***Clinical relevance*** *Management strategies should be adapted to the type and severity of the reaction, as well as the urgency of required re-administration. Changing from the culprit contrast agent to another molecule with differing side-chains is more effective than premedication.*

## Introduction

The Contrast Media Safety Committee (CMSC) of the European Society of Urogenital Radiology (ESUR) updated its guidelines on the prevention of hypersensitivity reactions (HR) to intravascular (intravenous (IV) or intra-arterial (IA)) administration of contrast media (CM).

To prevent a recurrent HR, it is essential to ascertain whether the patient has previously experienced a reaction following a contrast-enhanced examination, with established symptoms of an HR, according to the description included in Part I of this update [1], based on the American College of Radiology (ACR) Manual on Contrast Media [2]. Additionally, it is a priority to identify the specific CM involved. Unfortunately, the details of previous HR are frequently unavailable or inadequately documented. Therefore, it is recommended to implement a questionnaire to ensure that, among other possible risk factors, information about previous adverse reactions is accurately collected [3].

It has long been known that the evidence for administering premedication to prevent HR to CM is very weak [4, 5], and its use is recently getting reduced in multiple countries [6–8]. The role of the allergologist is expanding, and recently allergy assessments have shown to be cost-effective [9] and have considerably improved the prevention of recurrent HR to CM, allowing for selection of safe alternative CM for follow-up imaging [10, 11].

The purpose of Part 2 is to provide general recommendations on how to effectively manage a patient with a history of a HR to intravascular administration of CM, who is scheduled for a contrast-enhanced examination. Given the considerable variation in the availability of allergologists across Europe [12], as well as differences in the types of CM used, these recommendations aim to establish a framework for local protocols tailored to the specific circumstances of each institution.

The following aspects will be covered: In vitro tests to diagnose HR to CM, skin tests (ST), and drug provocation tests (DPT) to identify subjects with true hypersensitivity to one or more specific CM and to identify the safest alternative CM, and measures to prevent or minimize the risk of recurrent HR to CM.

## Methods

Three systematic literature analyses were repeatedly performed using PubMed and Embase databases from January 1985 until May 2022. Search criteria for in vitro tests included (synonyms of) “contrast media,” “hypersensitivity,” “tryptase,” “basophil activation test,” “lymphocyte transformation test” and “in vitro test.” Search criteria for skin and provocation testing included (synonyms of) “contrast media,” “hypersensitivity,” “skin test,” “skin prick test,” “patch test,” “intradermal test” and “drug provocation test.” Search criteria for preventive measures included (synonyms of) “contrast media,” “hypersensitivity,” “premedication,” “prophylaxis,” and “contrast agent change.” Languages were limited to English and German. Studies with pediatric populations (< 18 years) were excluded since there is very limited information available for this group, and the existing data do not differ significantly from those obtained in adults. For this reason, decisions for children are generally based on the same principles as those for adults, with weight-based dose adjustments and a recommendation to avoid the use of parenteral corticosteroids whenever possible, but for certain indications, higher CM doses per kg body weight and higher injection rates may be used. Cross-referencing was used when appropriate (see also Online Supplement 1).

The initial literature searches produced 282, 336, and 431 hits, respectively. After May 2022, literature was added using multiple non-systematic reviews. Ultimately, 12, 25, and 28 studies were included in this final review following full-text evaluation by two experienced authors (A.J.v.d.M., A.A.J.M.v.d.V.). This extensive literature review provided the base for the consecutive guideline development process following the CMSC Working Rules (www.esur.org). The concept guideline was discussed and agreed upon at the ESUR CMSC meetings in September 2023 in Rome (Italy) and September 2024 in Lisbon (Portugal).

## In vitro tests for the diagnosis of hypersensitivity reactions to contrast media

### Immediate or acute hypersensitivity reactions (IHR)

#### Tryptase

Histamine and tryptase are both biomarkers to confirm IHR. However, histamine is degraded quickly, is less specific, and is more difficult to measure using commercial assays [11, 13]. Elevated tryptase levels usually indicate IgE-mediated mast cell activation and mediator release, correlating with the clinical severity of the reaction [10, 14, 15]. In mild or moderate IHR, tryptase levels typically remain normal, and the absence of elevation does not exclude the possibility of a genuine IHR reaction [13].

According to the latest European Association of Allergy and Clinical Immunology (EAACI) practice guideline, measuring tryptase during the acute phase of a severe IHR is the most effective approach for confirming IHR, especially if a transient increase can be detected [11]. Ideally, three samples should be obtained: the first as early as possible during a suspected hypersensitivity reaction, the second at 1–2 h after the first but no later than 4 h after the onset of the reaction, and the third more than 24 h after all signs and symptoms have subsided [3, 16, 17]. The third sample evaluates the individual baseline level, helping in distinguishing between a constitutively increased tryptase level (e.g., mastocytosis or hereditary alpha-tryptasemia) and acute mast cell degranulation (tryptase levels significantly increased compared to baseline). An acute-over-baseline elevation of tryptase levels of at least 2 ng/mL + (1.2 × baseline tryptase level) during or within 4 h after symptoms is suggestive of an IHR [11, 18, 19].

#### Basophil activation test

The basophil activation test (BAT) is a flow-cytometry-based cellular assay that measures the upregulation of basophil activation markers following allergen stimulation [20]. BAT is valuable in diagnosing IHR to CM, especially in cases involving severe reactions or when ST or DPT are contraindicated and showing good correlation with ST and DPT results [21, 22]. Its specificity exceeds 90%, but its sensitivity is typically only slightly above 50% [23]. This low sensitivity is largely attributed to the limited expression of the mast cell receptor Mast-Related G-Protein-Coupled Receptor X2 (MRGPRX2) in basophils, which plays a key role in many non-IgE-mediated IHR to CM [21, 24, 25]. Furthermore, BAT is only available at specialized drug allergy centers.

### Non-immediate or delayed hypersensitivity reactions (NIHR)

#### Lymphocyte transformation test

The lymphocyte transformation test (LTT) should not be performed in the acute stage of NIHR, but 4–8 weeks after remission [26, 27]. LTT may be recommended as an additional diagnostic tool in selected cases when ST is contraindicated. High doses of corticosteroids and other immune-modulatory agents may interfere with LTT results. Its negative predictive value for NIHR is unknown. LTT is not widely available and should only be performed in experienced centers [11].

## In vivo tests for the diagnosis of hypersensitivity reactions to contrast media

### Skin tests

The presence of positive ST in patients with IHR and NIHR has been reported, indicating that immunological mechanisms are involved in these types of reactions [10, 21, 28]. In the case of IHR, between 15 and 58% of patients showed positive ST [14, 29–32], suggesting that these are more likely to be IgE-mediated allergic hypersensitivity reactions and carry a higher risk of developing more severe reactions [10, 33]. Intradermal tests (IDT) are more sensitive than skin prick tests (SPT) for diagnosis [32], and when an IDT with the culprit CM is negative, additional ST may not be necessary [34, 35]. The negative predictive value of ST is high, especially for IHR [21, 29, 36–39]. Therefore, when selecting an alternative CM for imaging, one with negative ST results should be preferentially used [11, 31, 40, 41].

All ST for the evaluation of HR to CM should adhere to EAACI standards [10, 27, 41, 42] and have been shown to be cost-effective [9]. ST should include the broadest possible panel of ICM or GBCA, including the culprit CM, if known. The sensitivity of ST is highest when performed within 2–6 months of the reaction, but decreases when performed within the first month or beyond 12 months of reaction [11]. ST should begin with SPT using undiluted CM and, if negative, proceed with IDT using 1:10 dilution [30, 43–45].

For NIHR, delayed IDT readings are the most sensitive diagnostic tool [46]. For severe cutaneous adverse reactions (SCAR), testing should be delayed for > 6 months, with patch test (PT) preferred over SPT and IDT since it imposes a lower risk of systemic reactions [44, 45]. PT is conducted with undiluted culprit CM, and readings are delayed for 48–96 h.

### Provocation tests

DPT, carefully administered by an allergologist with incremental CM doses, is a state-of-the-art allergological procedure. It is also the most potentially harmful and laborious procedure, available only in specialized centers for selected patients. DPT can be useful to verify the involvement of a CM in an HR and to confirm the tolerance of an alternative CM, especially when safe alternatives cannot be identified using ST or BAT [47]. Only CM with negative ST results should be administered.

Recently, several European groups have published promising results on the utility and safety of different DPT for CM [21, 37, 48–50]. A protocol for the rapid administration of CM with high flow rates has been reported, closely resembling its administration in imaging with ICM and GBCA [51, 52].

This approach may provide a better assessment of alternative mechanisms involved in HR to both ICM and GBCA [53–55], such as activation of the MRGPRX2-receptor. The activation of this mast cell receptor occurs independently of IgE or IgG involvement but requires a high concentration of the CM. Such high concentrations can only be achieved when the CM is administered at high doses and fast infusion rates, as used in these rapid DPT protocols. DPT for CM will likely assume a more prominent role in the future if conducted by experienced allergologists and with careful selection of patients using ST.

## Preventive measures in hypersensitivity reactions to contrast media

### Alternative imaging modality

In patients with a previous history of HR to a CM, an alternative imaging modality that does not require CM of a similar class (e.g., GBCA instead of ICM) or performing the examination without a CM should be considered, but only if the diagnostic yield is sufficient for correct patient management. The decision to omit a CM is more compelling when the HR was severe, but the patient should never be denied a well-indicated examination if a diagnostic alternative is not available. For milder HR and when alternative imaging modalities result in substantially lower quality, opting for an alternative imaging modality is less pertinent. In all cases, communication with the referring specialist is recommended.

### Use of premedication

In premedication, corticosteroid monotherapy was historically used in studies using high-osmolar CM (HOCM) [56], but these results cannot be extrapolated to current practice using low-osmolar (LOCM). There are no comparative randomized trials available, and several studies indicate that corticosteroids provide no additional benefit in preventing a recurrent HR [57, 58]. This is supported by a large retrospective study showing that patients who received a different ICM had a significantly lower rate of recurrent HR compared to those who received the same ICM with corticosteroid premedication (same ICM and steroid premedication: 80 of 423 examinations (19%); different ICM and no steroid premedication: 10 of 322 examinations (3%); odds ratio (OR), 0.14 [95% confidence interval (CI): 0.06, 0.33]; *p* < 0.001) [59]. Therefore, a recent anaphylaxis guideline from two American Allergology Societies concluded that evidence was lacking to support corticosteroid premedication [60]. Corticosteroid premedication can transiently elevate blood glucose [61] and may also be associated with prolonged hospital stays, increased costs, and worse outcomes [62].

H1-antihistamine monotherapy has been successfully used in mild HR in Korea [57, 63, 64]. However, these findings may be biased as individuals with milder reactions usually did not receive premedication [57, 65].

More frequently, two drugs are typically employed: H1-antihistamines and corticosteroids (see also Part 1 [1]). Frequently, they are administered together, which complicates the assessment of their individual effects, especially given the variations in premedication protocols. Traditional protocols from the era of HOCM [56, 66, 67] and early days of LOCM [68] continue to be widely utilized, including shorter intravenous options for hospitalized patients [69]. The utility of premedication is lower in NIHR [11, 63, 68], and the EAACI guideline does not recommend premedication for NIHR [11].

The evidence regarding the efficacy of corticosteroids and H1-antihistamines for prevention is highly heterogeneous and of low quality [4, 5]. Premedication may decrease the incidence of HR, mainly by reducing the number of mild reactions and thus the overall number of HR [67, 68] but does not significantly impact severe HR [70].

Several studies have investigated combination premedication with H1-antihistamines and corticosteroids. These regimens are frequently stratified according to the severity of the previous HR, with H1-antihistamines only prescribed for mild HR and H1-antihistamines plus corticosteroids for moderate-to-severe HR [57, 65]. Alternatively, these protocols have been adapted according to clinical preference.

Although there is less data on the effectiveness of premedication prior to GBCA use, the few available studies show similar results. H1-antihistamines may reduce the risk in milder HR, but it remains uncertain whether they also reduce or ameliorate symptoms in moderate-to-severe HR, even when administered in combination with corticosteroids [5, 71–73]. It is known that the affinity of histamine is higher than the affinity of antihistamines to H1-receptors, which may partly explain this effect [74].

**Fig. 1:**
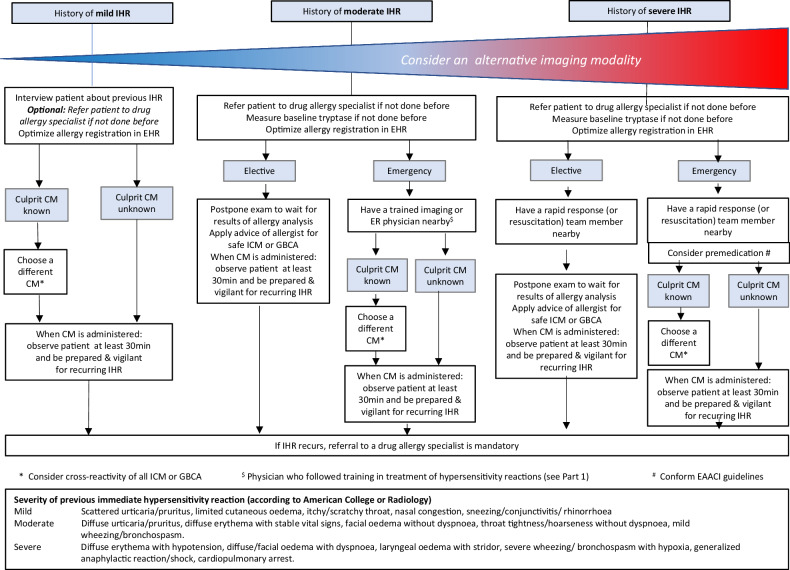
What to do after a previous immediate hypersensitivity reaction (IHR). # Conform EAACI guidelines: Torres et al [11]. CM, contrast medium/media; EHR, electronic health record; GBCA, gadolinium-based contrast agent; ICM, iodine-based contrast medium; IHR, immediate hypersensitivity reaction

In summary, there is no evidence that premedication reduces the risk of severe IHR to CM. Evidence for its role in previous mild to moderate IHR remains weak and conflicting, and premedication is not recommended for previous NIHR. Therefore, the CMSC, along with other major international guidelines [3, 6, 8, 11], has decided not to recommend routine premedication. Premedication is optional in emergency situations where an unidentified culprit CM led to a severe HR, as recommended by the EAACI [11] (Fig 1).

### Changing the specific contrast medium

Recently, changing the culprit CM has emerged as a viable preventive strategy, proving to be a superior alternative to premedication. Comparative studies have shown that an alternative ICM was more effective than premedication in preventing HR [57, 59, 63, 75–79]. For example, in a multivariate analysis it was shown that the risk of recurrent HR was 67.1% lower in cases where the implicated ICM was changed to another one (OR: 0.329; 95% CI, 0.168–0.644, *p* = 0.001), while steroid premedication did not significantly affect risk of recurrent HR (OR 0.907; 95% CI, 0.429–1.918, *p* = 0.799) [63]. Data on changing GBCA is more limited but shows a similar decrease in the rate of HR [71, 80, 81], with odds ratios for recurrent allergic-like HR of 0.7 (*p* = 0.041) after premedication and 0.2 (*p* < 0.001) after changing the type of GBCA in a large retrospective study [80]. Switching CM appears to be particularly useful in moderate-to-severe hypersensitivity reactions (OR, 0.30; 95% CI: 0.16–0.55; *p* < 0.001) compared to mild reactions (OR, 0.51; 95% CI: 0.37–0.69; *p* < 0.001) [77]. HR are not triggered by iodine or gadolinium atoms; instead, they are induced by specific epitopes present in one or more CM. The exact region of the CM molecule that acts as an epitope has not been identified, although it is generally considered to be located in the carbamoyl sidechain [82]. It is also possible that, in some HR, particularly delayed-type reactions, CM may act as a hapten requiring an appropriate carrier to function as a complete allergen [83]. Thus, switching to a CM that lacks this epitope or hinders its binding to the carrier could help prevent a recurrent HR. However, since the exact molecular region of CM capable of inducing an HR remains unidentified, a switch based on practical experience may not entirely prevent a new reaction, mainly due to the high and variable cross-reactivity (CR) between molecules. In this guideline, the term CR is used when patients experience an HR following exposure to two or more different CM or when there are positive ST for two or more CM. When using ionic CM, ions like meglumine may also cause HR [55, 84]. Furthermore, certain excipients of CM, like trometamol, may very rarely cause HR [85].

### Changing contrast media guided by a prior allergy evaluation

The CMSC recommends referring at least all patients who experienced moderate or severe HR to the allergologist for assessment. Optionally, all HR can be referred in areas with sufficient drug allergy specialist capacity.

The safest method for switching CM involves defining an alternative CM guided by an allergy evaluation. In a meta-analysis on the efficacy of ST, it was proposed that ST could serve as a potentially valuable diagnostic tool to confirm reactive ICM and help in identifying alternative ICM without CR [86]. This capacity of ST to identify safe alternative CM in patients with a history of IHR has been confirmed [30, 31]. Therefore, CM with negative ST results are highly likely to be safe alternatives. CR patterns could be established, especially when the culprit is known and shows positive ST results. However, establishing CR patterns is challenging if the culprit ST remains negative, as HR may still recur after administration of alternative ICM with negative ST [39, 50, 86]. DPT should then be considered [11, 47, 50].

### Change of contrast media based on practical experience

Given the varying levels of access to drug allergy specialists across Europe [12], situations may arise where timely allergy analysis is not possible, particularly in emergency or inpatient imaging.

The potential high reactivity of CM complicates a change based on practical experience. Several studies have attempted to assess the CR between ICM, but their findings are contradictory, making it difficult to draw clear conclusions [30, 31, 86–89]. Switching to an iso-osmolar ICM may significantly increase the risk of inducing NIHR [11], especially between iohexol and its dimeric form iodixanol [30, 31, 86]. The 2D chemical distinctions between different non-ionic ICM seem insufficient for deducing their level of CR. In various classifications, one group sharing a common N-(2,3-dihydroxypropyl)-carbamoyl sidechain is defined [30, 87–90], while other authors discriminate another group sharing an N-(2,3-dihydroxypropyl)-N-methyl-carbamoyl sidechain and a residual group lacking these carbamoyl sidechains [89–91]. The carbamoyl sidechain appears to be relevant, and a change from a CM carrying this sidechain to one without reduced subsequent reactions, whereas a change to an agent with a similar sidechain may be insufficient [22, 77–79, 92]. It cannot be inferred from these classifications that CR within the same group is complete, nor that in the event of a HR to an ICM from one group, an ICM from another group can be safely used. CR is more complex, with possible relevance to the 3D conformation of the epitope as uncommon associations in CR were seen after IHR between ioxitalamate and amidotrizoate (OR 7.7; 95% CI, 1.18–49.84) and after NIHR between amidotrizoate and iopamidol (OR 20.9; 95% CI, 2.21–197.5) and amidotrizoate and ioxitalamate (OR 21.4; 95% CI, 2.26–201.71) [91]. Of note, several studies reported greater tolerance for iobitridol when it is chosen based on practical experience as an alternative ICM, and a lower risk of ST positivity for iobitridol in case of positive ST for iomeprol (OR 0.2; 95% CI 0.06–0.85) [91], although it remains to be determined if this is related to the presence of N-(2,3-dihydroxypropyl)-N-methyl-carbamoyl sidechains [93–95].

Regarding GBCA, the knowledge on CR is limited as most studies are retrospective, involve small populations, and utilize heterogeneous methodologies. The recurrence rate of HR decreases by 75% when a GBCA with a chelate with a different molecular structure is administered [71], with greater safety observed when using an alternative ST-negative GBCA [40, 41].

CR between linear and macrocyclic GBCA appears to be very low [10, 41], while CR seems to be higher among macrocyclic GBCAs [32, 33, 96, 97]. Consequently, a decision after HR to a macrocyclic GBCA would be to recommend a linear GBCA. However, this option has been complicated by the current European restriction on the use of linear GCBA, related to concerns about their risk for nephrogenic systemic fibrosis and long-term gadolinium deposition. DPT would facilitate the selection of an alternative GBCA [32, 47, 52].

**Fig. 2:**
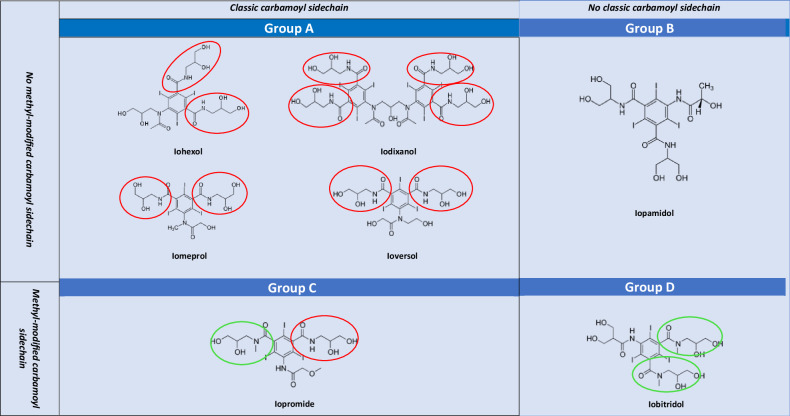
Chemical formulas and comparison of non-ionic ICM molecules and benzene chain arrangements by colors. Position 2, 4, 6 of the benzene ring: three iodine atoms (in all non-ionic ICM). Position 5 of the benzene ring: highly variable side chains defining diverse types of ICM (in all non-ionic ICM). Position 1 and 3 of the benzene ring Group A ICM (iohexol, iodixanol, iomeprol, ioversol): two classic carbamoyl sidechains (red). Group B ICM (iopamidol: two propane-based carbamoyl sidechains. Group C ICM (iopromide): one classic carbamoyl sidechain (red) and one methyl-modified carbamoyl sidechain (green). Group D ICM (iobitridol): two methyl-modified carbamoyl sidechains (green). Red Circle = N-(2,3-dihydroxypropyl)-carbamoyl sidechain (classic carbamoyl). Green Circle = N-(2,3-dihydroxypropyl)-N-methyl-carbamoyl sidechain.

**Fig. 3:**
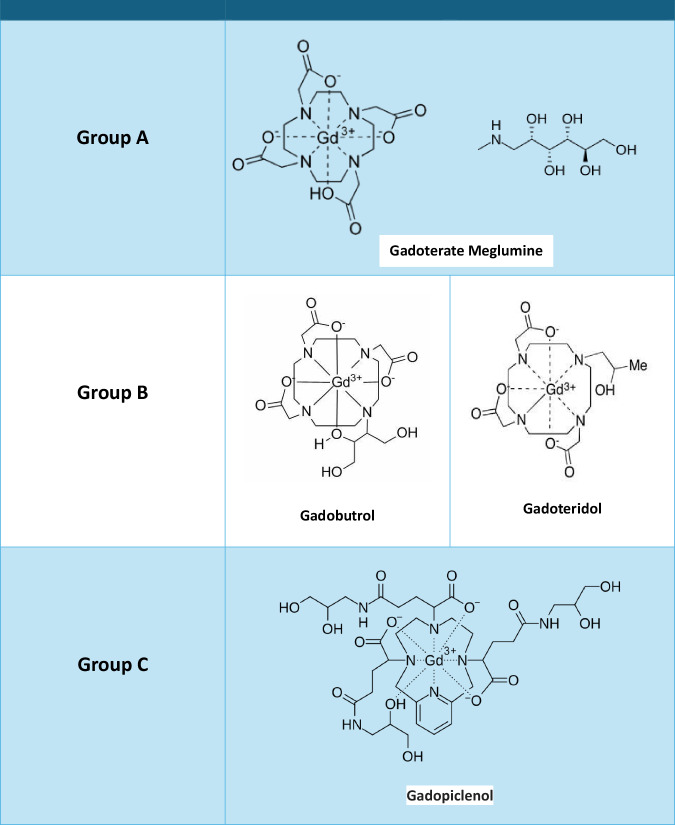
Chemical formulas and grouping of general, macrocyclic GBCA molecules. Group A: The chelating agent is DOTA (1,4,7,10-Tetraazacyclododecane-1,4,7,10-tetraaryl-tetraacetic acid). Includes gadoterate meglumine. Group B: The chelating agents are variants of DO3A (trisodium 1,4,7,10-tetraazacyclododecane-1,4,7-triacetate). Includes gadobutrol (with BT-DO3A) and gadoteridol (with HP-DO3A). Group C: The chelating agent is pyclen (3,6,9,15-tetraazabicyclo[9.3.1]pentadeca-1(15),11,13-triene -3,6,9-triacetic acid). Includes gadopiclenol

Regardless, changing CM based on practical experience remains inferior to allergological evaluation. Most studies on CR do not allow the choice of the safest CM on a robust scientific basis, with varying [31, 59, 77–79, 87, 88, 92, 93, 98]. A new, non-validated classification of ICM (Fig. 2) and GBCA (Fig. 3) has recently been published, based on previous clinical data on CM tolerance and integrating 2D and 3D conformation. This classification also includes suggestions regarding the selection of an alternative CM (Table 1) [90] but remains optional. However, the CMSC is currently unable to provide definitive evidence-based recommendations on how to change CM based solely on practical experience. In situations without allergic test support, early recognition of an IHR is important and physicians should use their available choices of alternative CM, adhere to local/national guidelines [6–8, 99], and provide that patients get optimal surveillance by staff trained in treatment of HR to CM, if needed with rapid response team (anesthesiologist + anesthesiology nurse) support (see Part 1 [1]).

**Table 1:** Suggestions regarding the selection of alternative contrast media based on practical experience

Iodine-based contrast media (ICM)	
Involved ICM	Suggestion regarding the selection of alternative ICM based on clinical practice reports [82–84]
***When the involved ICM is known***	
ICM is from Group A	Alternative ICM from Group B or D (without classic carbamoyl sidechain) *High cross-reactivity between Group A-Group C*
ICM is from Group B	Alternative ICM from Group A, C or D
ICM is from Group C	Alternative ICM from group B (without classic or methyl-modified carbamoyl sidechain)
ICM is from Group D	Alternative ICM from Group A or B (without methyl-modified carbamoyl sidechain)
***When the involved ICM is unknown***	
Due to the higher likelihood that the involved ICM is from group A	Choose the alternative ICM from Group B or D *High cross-reactivity between Group C-Group A*

Gadolinium-based contrast agents (GBCA)	
Involved GBCA	Suggestion regarding the selection of alternative GBCA based on practical experience
***When the involved GBCA is known***	
GBCA is from Group A	Alternative GBCA from Group B
GBCA is from Group B	Alternative GBCA from Group A
GBCA is from Group C	*Insufficient data for empiric change advice*
***When the involved GBCA is unknown***	
It is not possible to recommend a regimen with certainty. Due to the probability of involvement, using a GBCA different from the one routinely administered is suggested.	

Group A ICM: iohexol, iodixanol, iomeprol, ioversol

Group B ICM: iopamidol

Group C ICM: iopromide

Group D ICM: iobitridol

Group A GBCA: gadoterate meglumine

Group B GBCA: gadoteridol, gadobutrol

Group C GBCA: gadopiclenol

*GBCA* gadolinium-based contrast agent, *ICM* iodine-based contrast medium

## Conclusion

**Fig. 4:**
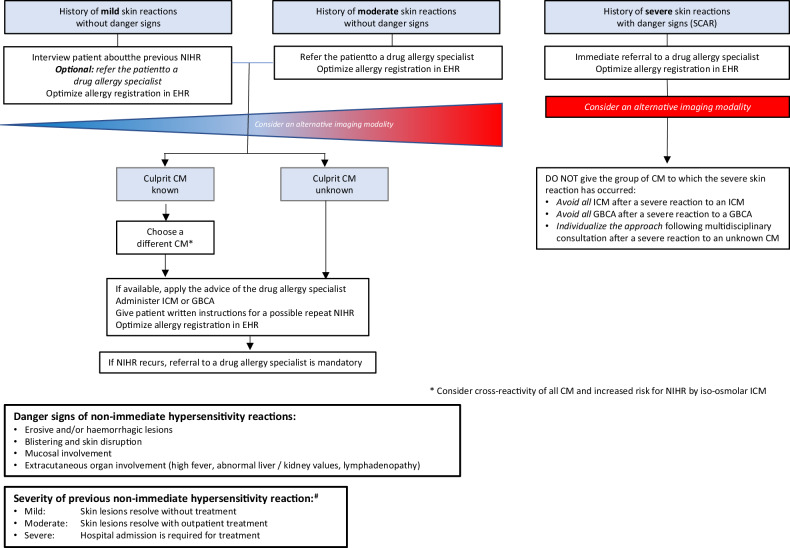
What to do after a previous non-immediate hypersensitivity reaction (NIHR). # Classification taken from Brockow et al [28]. CM, contrast medium/media; EHR, electronic health record; GBCA, gadolinium-based contrast agent; ICM, iodine-based contrast medium; NIHR, non-immediate hypersensitivity reaction; SCAR, severe cutaneous adverse reaction

One of the prime challenges in managing an HR to CM lies in selecting the most suitable preventive measure to prevent further reactions. Premedication is no longer considered protective, and recent studies advocate using an alternative CM, selecting the safest CM by allergy assessment as the optimal approach. Timely referral to allergologists may prevent complex situations when patients with a severe previous HR to an unknown CM are in immediate need of CM administration. Since these cases are complicated, an individualized approach based on the patient’s characteristics, indication for CM administration, and local preferences is recommended. Practical CMSC recommendations on the above topics have been summarized in Table 2 and Figs. 1 and 4, which can be adapted by national medical societies to the characteristics of their healthcare systems.

**Table 2:** CMSC recommendations for the prevention of recurrent hypersensitivity reactions in adults

**In vitro tests**
Measure serum tryptase within 1–4 h from the start of all moderate-to-severe immediate hypersensitivity reactions to contrast media. A second measurement after ≥ 24 h serves as a baseline for further allergologic examinations.
Reserve basophil activation tests for selected patients with moderate-to-severe acute hypersensitivity reactions, as they are only available in specialized drug allergy centers.
For non-immediate hypersensitivity reactions, there are no meaningful in vitro diagnostic tests in clinical use.
**Allergy assessment**
Refer the following patients to a drug allergy specialist for an allergy assessment:
• Patients experiencing moderate and severe hypersensitivity reactions, and optionally, patients experiencing mild hypersensitivity reactions when the drug allergy specialist capacity is sufficient • Patients experiencing hypersensitivity reactions to multiple iodine-based or gadolinium-based contrast agents (either two or more different iodine-based contrast media or gadolinium-based contrast agents or to an iodine-based contrast medium and a gadolinium-based contrast agent). • Patients experiencing recurrent hypersensitivity reactions of any severity. During the allergy assessment, test for the suspected culprit contrast agent and several commonly used alternatives, ideally within 6 months after the hypersensitivity reaction. See also Figs. 1 and 4 Always specify the used contrast medium in the referral to the drug allergy specialist. A detailed documentation about the culprit contrast agent and the severity of the reaction, including a grading scheme, is mandatory (see Part 1).
**Preventive measures**
In all patients with a history of a hypersensitivity reaction to an iodine-based contrast medium or a gadolinium-based contrast agent, consider an alternative imaging modality, or consider performing an unenhanced exam if the diagnostic yield is sufficient for the correct management of the patient. Never deny a patient a clinically well-indicated enhanced examination if alternative imaging strategies are not available. See also Figs. 1 and 4
*Immediate hypersensitivity reactions (IHR)*
In patients with a history of a **mild** immediate hypersensitivity reaction to an iodine-based contrast medium or a gadolinium-based contrast agent:
• Interview the patient about their previous hypersensitivity reaction • Optionally, refer the patient to a drug allergy specialist (if not done before) when the local drug allergy specialist capacity is sufficient • Optimize the allergy registration in the electronic health record • Apply the advice of the drug allergy specialist for a safe iodine-based contrast medium or gadolinium-based contrast agent, or, when not available, choose a different iodine-based contrast medium or gadolinium-based contrast agent if the culprit contrast agent is known* • When the contrast medium is administered, observe the patient for at least 30 min with the IV line in place • Be prepared and vigilant for a recurring immediate hypersensitivity reaction • If an immediate hypersensitivity reaction recurs, referral to a drug allergy specialist is mandatory * See also Fig. 1
In patients with a history of a **moderate** immediate hypersensitivity reaction to an iodine-based contrast medium or gadolinium-based contrast agent:
• Refer the patient to a drug allergy specialist (if not done before) • Optimize the allergy registration in the electronic health record
For **elective** contrast-enhanced examinations:
• Postpone imaging to wait for the results of the allergy analysis • Apply the advice of the drug allergy specialist for a safe iodine-based contrast medium or gadolinium-based contrast agent • When the contrast medium is administered, observe the patient for at least 30 min with the IV line in place • Be prepared and vigilant for a recurring immediate hypersensitivity reaction • If an immediate hypersensitivity reaction recurs, referral to a drug allergy specialist is mandatory
For **emergency** contrast-enhanced examinations:
• Have a trained imaging or emergency room physician nearby • Choose a different iodine-based contrast medium or gadolinium-based contrast agent if the culprit contrast medium is known* • When the contrast medium is administered, observe the patient for at least 30 min with the IV line in place • Be prepared and vigilant for a recurring immediate hypersensitivity reaction • If an immediate hypersensitivity reaction recurs, referral to a drug allergy specialist is mandatory * See also Fig. 1
In patients with a history of a **severe** immediate hypersensitivity reaction to an iodine-based contrast medium or gadolinium-based contrast agent:
• Refer the patient to a drug allergy specialist (if not done before) • Optimize the allergy registration in the electronic health record
For **elective** contrast-enhanced examinations:
• Have a trained rapid response (or resuscitation) team member nearby • Postpone imaging to wait for the results of the allergy analysis • Apply the advice of the drug allergy specialist for a safe iodine-based contrast medium or gadolinium-based contrast agent • When the contrast medium is administered, observe the patient for at least 30 min with the IV line in place • Be prepared and vigilant for a recurring immediate hypersensitivity reaction • If an immediate hypersensitivity reaction recurs, referral to a drug allergy specialist is mandatory
For **emergency** contrast-enhanced examinations:
• Have a trained rapid response (or resuscitation) team member nearby • Consider administration of premedication (EAACI guidelines) • Choose a different iodine-based contrast medium or gadolinium-based contrast agent if the culprit contrast medium is known* • When the contrast medium is administered, observe the patient for at least 30 min with the IV line in place • Be prepared and vigilant for a recurring immediate hypersensitivity reaction • If an immediate hypersensitivity reaction recurs, referral to a drug allergy specialist is mandatory * See also Fig. 1
Emergency premedication protocol:
• 50 mg prednisolone IV (or equivalent): ≥ 30 min before contrast medium administration • 2 mg clemastine IV (or equivalent): ≥ 30 min before contrast medium administration
Note: 50 mg prednisolone IV is equivalent to:
• 40 mg methylprednisolone IV • 8 mg dexamethasone IV • 200 mg hydrocortisone IV
Note: 2 mg clemastine IV is equivalent to:
• 50 mg diphenhydramine IV • 20 mg chlorphenamine IV • 10 mg cetirizine IV
*Non-immediate hypersensitivity reactions (NIHR)*
In patients with a history of a **mild** non-immediate hypersensitivity reaction to an iodine-based contrast medium or a gadolinium-based contrast agent without danger signs:
• Interview the patient about their previous hypersensitivity reaction • Optionally, refer the patient to a drug allergy specialist (if not done before) when the local drug allergy specialist capacity is sufficient • Optimize the allergy registration in the electronic health record • Apply the advice of the drug allergy specialist for a safe iodine-based contrast medium or gadolinium-based contrast agent, or, when not available, choose a different Iodine-based contrast medium or gadolinium-based contrast agent if the culprit contrast agent is known* • When the contrast medium is administered, observe the patient for at least 30 min with the IV line in place • Give the patient written instructions for a possible repeat non-immediate hypersensitivity reaction • If a non-immediate hypersensitivity reaction recurs, referral to a drug allergy specialist is mandatory * See also Fig. 4
In patients with a history of a **moderate** non-immediate hypersensitivity reaction to an iodine-based contrast medium or a gadolinium-based contrast agent without danger signs:
• Refer the patient to a drug allergy specialist (if not done before) • Optimize the allergy registration in the electronic health record • Apply the advice of the drug allergy specialist for a safe iodine-based contrast medium or gadolinium-based contrast agent, or, when not available, choose a different Iodine-based contrast medium or gadolinium-based contrast agent if the culprit contrast agent is known* • When the contrast medium is administered, observe the patient for at least 30 min with the IV line in place • Give the patient written instructions for a possible repeat non-immediate hypersensitivity reaction • If a non-immediate hypersensitivity reaction recurs, referral to a drug allergy specialist is mandatory * See also Fig. 4
In patients with a history of a **severe** non-immediate hypersensitivity reaction to an iodine-based contrast medium or a gadolinium-based contrast agent with danger signs** (SCAR): • Refer the patient immediately to a drug allergy specialist (if not done before) • Choose an alternative imaging modality • Optimize the allergy registration in the electronic health record DO NOT give the group of contrast media to which the severe skin reaction has occurred: • Avoid all iodine-based contrast media after a severe non-immediate hypersensitivity reaction to an iodine-based contrast medium • Avoid all gadolinium-based contrast agents after a severe non-immediate hypersensitivity reaction to a gadolinium-based contrast agent • Individualize the approach following multidisciplinary consultation after a severe reaction to an unknown CM See also Fig. 4
* Consider cross-reactivity of contrast media and an increased risk for non-immediate hypersensitivity reaction with the use of iso-osmolar dimeric iodine-based contrast media
** Danger signs: erosive and/or hemorrhagic lesions, blistering and skin disruption, mucosal involvement, extracutaneous organ involvement (high fever, abnormal liver/kidney values, lymphadenopathy)
**Cross-reactivity between contrast agents**
Cross-reactivity is most relevant in allergic hypersensitivity reactions and cannot be predicted on the basis of the chemical structure
Based on current knowledge, it can occur with a higher frequency among:
• Iodine-based contrast media with a N-(2,3-hydroxypropyl)-carbamoyl sidechain* • Macrocyclic gadolinium-based contrast agents * See Figs. 2 and 3
The drug allergy specialist determines through an evaluation, including skin testing with a panel of different iodine-based contrast media and gadolinium-based contrast agents:
• The allergic nature of the hypersensitivity reaction
• Cross-reactivity between contrast media • Suggestions for safe alternative contrast media
**Change to an alternative contrast agent based on practical experience**
CMSC cannot make evidence-based recommendations on a robust scientific basis for change to an alternative contrast agent based on practical experience. Imaging physicians should use their available choices of alternative contrast media, adhere to local or national guidelines, and ensure that patients get optimal surveillance by adequately trained staff, if needed, with rapid response (or resuscitation) team support.

*CMSC* Contrast Medium Safety Committee, *SCAR* severe cutaneous adverse reaction

## Supplementary information

ELECTRONIC SUPPLEMENTARY MATERIAL

## Abbreviations

BAT: Basophil activation test
CM: Contrast medium/media
CMSC: ESUR Contrast Media Safety Committee
CR: Cross-reactivity
DPT: Drug provocation test/tests
EAACI: European Association of Allergy & Clinical Immunology
ESUR: European Society of Urogenital Radiology
GBCA: Gadolinium-based contrast agent/agents
HOCM: High-osmolar contrast medium/media
HR: Hypersensitivity reaction/reactions
ICM: Iodine-based contrast medium/media
IDT: Intradermal test/tests
IHR: Immediate hypersensitivity reaction
LOCM: Low-osmolar contrast medium/media
LTT: Lymphocyte transformation test/tests
MRGPRX2: Mas-related G-protein-coupled receptor X2
NIHR: Non-immediate hypersensitivity reaction
OR: Odds ratio
PT: Patch test/tests
SCAR: Severe cutaneous adverse reaction/reactions
SPT: Skin prick test/tests
ST: Skin test/tests
