# Supplementary material for: Hypersensitivity reactions to contrast media: Part 2. Prevention of recurrent hypersensitivity reactions in adults. Updated guidelines by the ESUR Contrast Media Safety Committee
Source: Eur Radiol. 2025 May 27;35(11):6811–25. doi: 10.1007/s00330-025-11676-0 (PMC12559128; doi:10.1007/s00330-025-11676-0)
Supplement: Supplementary file 1 — ELECTRONIC SUPPLEMENTARY MATERIAL [file 330_2025_11676_MOESM1_ESM.pdf]

# Hypersensitivity Reactions to Contrast Media Part 2: Prevention of Recurrent Hypersensitivity Reactions in Adults. Updated Guidelines by the ESUR Contrast Media Safety Committee

## ELECTRONIC SUPPLEMENTARY MATERIAL

Eur Radiol (2025) van der Molen AJ, van de Ven AAJM, Vega FF, et al.

**Online Supplement 1:** Original literature search

*Topics:*
*In Vitro Tests in Patients with Hypersensitivity Reactions to Contrast Media*
*Diagnostic Value of Skin Testing for Hypersensitivity Reactions to Contrast Media*
*Prophylactic Measures for Prevention of Recurrent Hypersensitivity Reactions to Contrast Media*

*Joint Search strategy*

| Database | Zoektermen |
|---|---|
| PubMed<br><br>1985 – May 2022 | ((("Contrast Media"[Mesh] OR contrast medi* [tiab] OR contrast agent* [tiab] OR contrast material* [tiab] OR contrast dose [tiab] OR contrast doses [tiab] OR contrast dosage [tiab] OR radiocontrast medi* [tiab] OR radiocontrast agent* [tiab] OR radiopaque medi* [tiab] OR radiocontrast dose [tiab] OR radiocontrast doses [tiab] OR radiocontrast dosage [tiab] OR "Barium"[Mesh] OR barium [tiab] OR gadolinium [tiab] OR microbubble* [tiab]) AND ("Drug Hypersensitivity"[Mesh] OR hypersensitiv* [tiab] OR allerg* [tiab] OR anaphyla* [tiab] OR "Exanthema"[Mesh] OR exanthem* [tiab] OR rash [tiab] OR adverse reaction*[tiab] OR drug reaction* [tiab] OR urticaria* [tiab] OR erythem* [tiab] OR edema [tiab] OR angioedema [tiab] OR bronchospasm* [tiab] OR hypotension [tiab] OR hypertension [tiab] OR cardiac arrest* [tiab] OR respiratory arrest [tiab] OR "Stevens-Johnson Syndrome"[Mesh] OR stevens johnson syndrome [tiab] OR sjs [tiab] OR toxic epidermal necrolys* [tiab] OR "Drug Hypersensitivity Syndrome"[Mesh] OR dress syndrome [tiab] OR iodide mump* [tiab] OR ((late [tiab] OR delayed [tiab] OR nonimmediate [tiab] OR immediate [tiab] OR acute [tiab] OR severe [tiab]) AND (reaction* [tiab])))<br><br>AND (serum hypersensitivity test* [tiab] OR "Immunoglobulin E"[Mesh] OR IgE [tiab] OR "Tryptases"[Mesh] OR tryptase* [tiab] OR urinary histamine metabolite* [tiab] OR "Methylhistamines"[Mesh] OR methylhistamine* [tiab] OR methylimidazole acetic acid* [tiab] OR basophil activation test* [tiab]))<br><br>AND (("english"[Language]) AND ("1985"[Date - Publication] : "3000"[Date - Publication])) |
| Embase | (('contrast medium'/exp OR (((contrast OR radiocontrast) NEAR/2 (medi* OR agent* OR material* OR dose OR doses OR dosage)):ab,ti) OR 'radiopaque medi*':ab,ti OR 'barium'/exp OR barium:ab,ti OR 'gadolinium'/exp OR gadolinium:ab,ti OR 'microbubble'/exp OR microbubble*:ab,ti)<br><br>AND ('hypersensitivity'/exp OR hypersensitiv*:ab,ti OR anaphyla*:ab,ti OR allerg*:ab,ti OR 'rash'/exp OR rash:ab,ti OR 'adverse reaction*':ab,ti OR 'drug reaction*':ab,ti OR urticaria*:ab,ti OR erythem*:ab,ti OR exanthem*:ab,ti OR edema:ab,ti OR angioedema:ab,ti OR bronchospasm*:ab,ti OR 'anaphylactic shock':ab,ti OR hypotension:ab,ti OR hypertension:ab,ti OR 'cardiac arrest':ab,ti OR 'respiratory arrest':ab,ti OR 'stevens johnson syndrome'/exp OR 'stevens johnson syndrome':ab,ti OR sjs:ab,ti OR 'toxic epidermal necrolysis'/exp OR 'toxic epidermal necrolys*':ab,ti OR 'dress syndrome'/exp OR 'dress syndrome':ab,ti OR 'iodide mump*':ab,ti OR (((late OR delayed OR nonimmediate OR immediate OR acute OR severe) NEAR/2 reaction*):ab,ti))<br><br>AND ('serum hypersensitivity test*':ab,ti OR 'immunoglobulin E'/exp OR IgE:ab,ti OR 'tryptase'/exp OR tryptase*:ab,ti OR 'urinary histamine metabolite*':ab,ti OR 'methylhistamine'/exp OR methylhistamine*:ab,ti OR 'methylimidazole acetic acid*':ab,ti OR 'basophil activation test'/exp OR 'basophil activation test*':ab,ti))<br><br>AND [english]/lim AND [1985-2018]/py NOT 'conference abstract':it NOT (('animal experiment'/exp OR 'animal model'/exp OR 'nonhuman'/exp) NOT 'human'/exp) |
